# Sex differences in story recall decline in preclinical Alzheimer's disease

**DOI:** 10.1093/braincomms/fcaf169

**Published:** 2025-05-05

**Authors:** Douglas Cowman, Rebecca Langhough, Hayley Olson, Kristin Basche, Leah Sanson-Miles, Davide Bruno, Bruce Hermann, Bradley T Christian, Tobey J Betthauser, Sterling C Johnson, Kimberly D Mueller

**Affiliations:** Department of Communication Sciences and Disorders, University of Wisconsin—Madison, Madison, WI 53792, USA; Wisconsin Alzheimer’s Institute, University of Wisconsin School of Medicine and Public Health, Madison, WI 53792, USA; Division of Geriatrics and Gerontology, Department of Medicine, University of Wisconsin-Madison School of Medicine and Public Health, Madison, WI 53726, USA; Wisconsin Alzheimer’s Disease Research Center, University of Wisconsin School of Medicine and Public Health, Madison, WI 53792, USA; Department of Communication Sciences and Disorders, University of Wisconsin—Madison, Madison, WI 53792, USA; Division of Geriatrics and Gerontology, Department of Medicine, University of Wisconsin-Madison School of Medicine and Public Health, Madison, WI 53726, USA; Department of Communication Sciences and Disorders, University of Wisconsin—Madison, Madison, WI 53792, USA; School of Psychology, Liverpool John Moores University, Liverpool L3 3AF, UK; Wisconsin Alzheimer’s Institute, University of Wisconsin School of Medicine and Public Health, Madison, WI 53792, USA; Department of Neurology, University of Wisconsin School of Medicine and Public Health, University of Wisconsin—Madison, Madison, WI 53726, USA; Wisconsin Alzheimer’s Disease Research Center, University of Wisconsin School of Medicine and Public Health, Madison, WI 53792, USA; Waisman Laboratory for Brain Imaging and Behavior, University of Wisconsin—Madison, Madison, WI 53705, USA; Department of Medical Physics, University of Wisconsin—Madison, Madison, WI 53705, USA; Wisconsin Alzheimer’s Institute, University of Wisconsin School of Medicine and Public Health, Madison, WI 53792, USA; Wisconsin Alzheimer’s Disease Research Center, University of Wisconsin School of Medicine and Public Health, Madison, WI 53792, USA; Department of Medical Physics, University of Wisconsin—Madison, Madison, WI 53705, USA; Wisconsin Alzheimer’s Institute, University of Wisconsin School of Medicine and Public Health, Madison, WI 53792, USA; Wisconsin Alzheimer’s Disease Research Center, University of Wisconsin School of Medicine and Public Health, Madison, WI 53792, USA; Department of Communication Sciences and Disorders, University of Wisconsin—Madison, Madison, WI 53792, USA; Wisconsin Alzheimer’s Institute, University of Wisconsin School of Medicine and Public Health, Madison, WI 53792, USA; Wisconsin Alzheimer’s Disease Research Center, University of Wisconsin School of Medicine and Public Health, Madison, WI 53792, USA

**Keywords:** Alzheimer’s disease, sex, amyloid-beta, language, memory

## Abstract

Stage II pre-clinical Alzheimer's disease is defined by the presence of increased amyloid-beta evidenced by fluid and/or imaging biomarkers, in the absence of clinical signs and symptoms. Previous research suggests that pre-clinical sex differences exist on measures of story recall, such as the Wechsler memory scale-revised logical memory test total score. However, sex differences on a composite metric of proper names from that test have not been investigated, and the relationships between sex and amyloid positivity on longitudinal logical memory measures are unclear. We examined longitudinal trajectories of total score and proper names by sex (Aim 1), and by the combination of sex and amyloid status (Aim 2). *N* = 457 Wisconsin registry for Alzheimer's prevention participants with PET Pittsburgh compound B-assessed amyloid status (+/−) were included. Linear mixed-effects models were used to examine the interaction between sex and age at visit (the time variable), and sex and amyloid+/− on longitudinal total and proper name scores. Aim 1 analyses showed a main effect such that female participants, on average, scored higher than males on both total and proper name recall. The interaction between sex and age was not statistically significant, indicating that both sexes experienced a similar average rate of annual decline. Aim 2 analyses showed that amyloid positive participants, regardless of sex, showed steeper declines compared to amyloid negative, female participants (reference group). Thus, while female participants generally outperformed males on story recall measures, the impact of amyloid burden on longitudinal story recall trajectories was not significantly more pronounced in females. Results emphasize the need for further exploration into sex-specific cognitive reserve mechanisms in the context of Alzheimer's disease biomarker burden, as well as in the assessment and understanding of cognitive decline trajectories.

## Teaser

Cowman *et al*. report that females outperform males on story and proper name recall in cognitively unimpaired adults at risk for Alzheimer's disease. While amyloid positivity predicts faster decline on both measures, sex is not a moderator. Findings highlight the importance of novel measures and sex-specific mechanisms in Alzheimer's research.

## Graphical Abstract

**Graphical Abstract:**
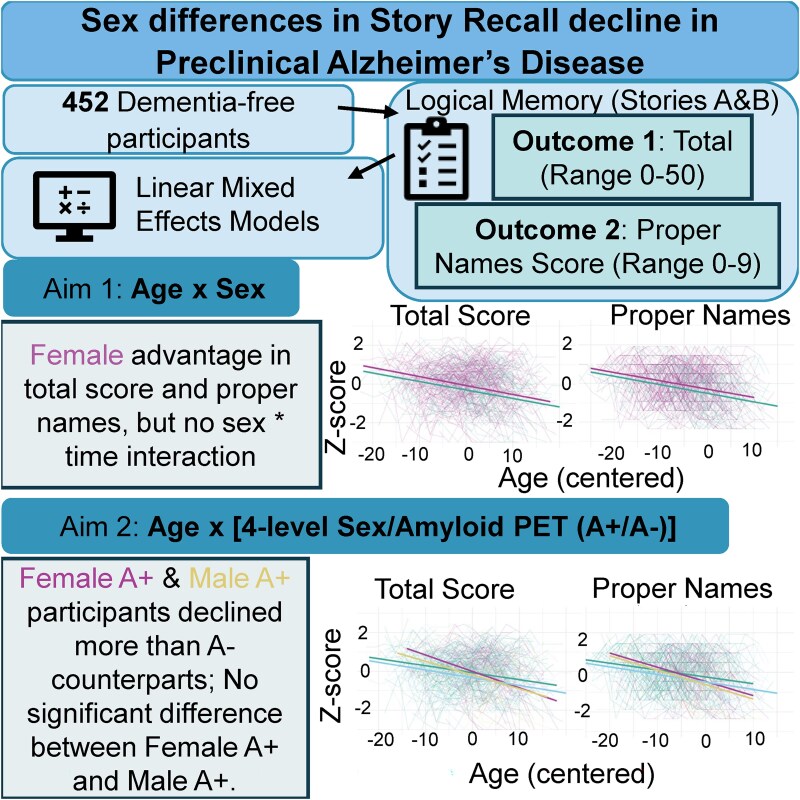

## Introduction

Pre-clinical Alzheimer's disease is defined by the presence of increased amyloid-beta (Aβ) evidenced by fluid and/or imaging biomarkers, in the absence of clinical signs and symptoms of the disease.^1^ Females account for approximately two-thirds of persons living with Alzheimer's disease in the USA and worldwide.^2,3^ While this is partly explained by differences in longevity between sexes, previous research suggests pre-clinical sex differences exist across some cognitive domains, particularly verbal learning and memory.^4,5^ For instance, in cognitively healthy persons throughout life, females have been consistently shown to outperform males on tests of verbal episodic memory.^6,7^ This purported female advantage is maintained in persons with amnestic mild cognitive impairment (MCI), but some have found it to be lost in individuals with Alzheimer's disease dementia, possibly due to different rates of decline in females with MCI than males.^2,4,5,8^ Conversely, others report that females continue to outperform males despite advancing disease burden.^9^ Females scoring higher on tests of verbal memory and subsequently losing this advantage in Alzheimer's disease dementia may infer a sex-specific cognitive reserve.^9^ The cognitive reserve theory hypothesis proposes that certain characteristics such as advanced education and above average IQ provide a reserve capacity of greater cognitive strategies and connections that can be maintained despite Alzheimer's disease pathology. It predicts that persons with greater reserve have more observed Alzheimer's disease pathology than those without at similar levels of clinical progression. Once brain pathology passes a threshold level, decline is more accelerated in those with high reserve due to more advanced pathology.^10-13^ The possibility of sex-specific cognitive reserve deserves better understanding since verbal memory tests are the main tools used to identify MCI and Alzheimer's disease dementia. These sex differences may delay an MCI diagnosis and mask underlying brain pathology in females.^14^

The aforementioned research has focused on ‘total scores’; that is, the sum of the verbal items recalled on a particular task, such as list-learning or story recall tasks. However, the most utilized tests, such as Logical Memory story recall from the Wechsler memory scale-revised (WMS-R), can be insensitive to subtle cognitive decline associated with pre-clinical Alzheimer's disease.^15^ Prior research has suggested that ‘process scores’, or the quantification of errors or other aspects of an individual's spontaneous recall performance (the process by which they arrived at the total score), may be more sensitive to subtle decline than total scores alone.^16^ For example, our group has examined the lexical categories of the words recalled in WMS-R logical memory story recall, and that the lexical category of ‘proper names’ (i.e. names of people and places) was uniquely and significantly sensitive to Alzheimer's disease biomarkers in a cognitively unimpaired sample of adults at increased risk for Alzheimer's disease. Specifically, proper names were associated with Aβ status, while none of the other story recall variables, including total score, were associated with Aβ status, and the proper names model was the model of best fit, suggesting that individual items in logical memory stories may be useful tools for early detection of cognitive decline.^17,18^

Previous research suggests that sex-specific norms for verbal memory tests may improve diagnostic accuracy of amnestic MCI.^17^ Sex differences on logical memory measures in the Wisconsin registry for Alzheimer's prevention (WRAP) study have not yet been examined. Whether sex differences on item-level story recall in logical memory exist and whether they are related to Alzheimer's disease biomarkers remains uncertain and needs to be understood to further the diagnostic toolbox for more accurate early detection. Sex-specific trajectories of decline on proper name recall are also yet to be researched.

We had two main aims: to determine whether the rate of decline on total and item level (proper names) logical memory delayed recall measures differ between sexes (Aim 1) and, to investigate whether rates of decline on these same logical memory measures differ by sex/PiB status group (Aim 2). We hypothesized that females would perform better at baseline and decline at a rate similar to males (Aim 1), and that amyloid positivity would moderate the effect of sex on LMT and PN longitudinal trajectories (Aim 2). To explore these questions, we used the logical memory story recall task from the WMS-R from the WRAP, a longitudinal cohort study of participants enriched for Alzheimer's disease risk who are free of dementia and predominantly cognitively unimpaired at cognitive baseline.^18^

## Materials and methods

### Participants

**Figure 1:**
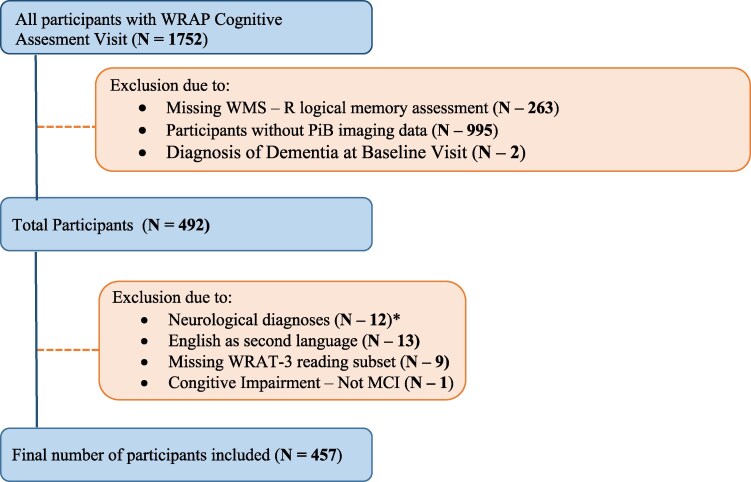
**Participant selection and exclusion process.** Inclusion and exclusion criteria process for participant selection from the WRAP sample. WMS-R, Weschler memory scale-revised; WRAP, Wisconsin registry for Alzheimer's prevention; PiB, Pittsburgh compound B; WRAT-3, wide range achievement test-3. *37 participants excluded due to previous diagnoses of meningitis (*N* = 4), stroke (*N* = 2), epilepsy (*N* = 1), and other neurological diagnoses (*N* = 5).

The study sample was drawn from the WRAP study, utilizing data from the May 2022 WRAP data freeze. WRAP is an ongoing longitudinal observational cohort of participants who enrolled in early to late middle age and were non-demented at baseline; in addition, the sample is enriched for Alzheimer's disease risk due to ∼75% of participants having a parental history of Alzheimer's disease.^19^ The second visit occurred 2-4 years after the baseline, and subsequent visits occurred every 2 years after the first follow-up (see Johnson *et al*.^19^). WMS-R logical memory was first added to the cognitive test battery in 2007, and item-level data was first entered in summer 2018, including available retrospective data from previous cognitive visits.^18^ Logical memory baseline is defined as the first visit in which the WMS-R logical memory recall task was administered to each participant (median visit = 2). Participants were included in the present analyses from the WRAP cohort if at the time of these analyses they had participated in logical memory assessments and Alzheimer's disease biomarker studies, were free of neurological diagnoses at any visit, were dementia free at logical memory baseline, and had English as their first language (Fig. 1). To focus on longitudinal trajectories, participants were required to have at least two follow-up visits. This ensured that all included data were sufficiently robust for modelling longitudinal changes over time.

### Helsinki declaration

Human subjects participation in these studies was approved by the University of Wisconsin Health Sciences Institutional Review Board, in accordance with the Helsinki declaration. All participants provided informed consent.

### Cognitive assessment battery

Participants underwent a comprehensive neuropsychological battery at each WRAP visit. For this analysis, tests were selected that measure memory, language, or global cognitive function. To characterize the study sample, we included WMS-R logical memory I and II (immediate and delayed recall, respectively); wide range achievement test—3rd edition reading subtest (WRAT-3); and mini mental state examination (MMSE).^19^ We included MMSE to help characterize the sample due to WRAP being mostly healthy at the baseline logical memory test.

### Story recall outcomes

Standard procedures for the logical memory test administration were followed in accordance with the WMS-R manual.^19^ In this task, two short stories (Story A and Story B) made up of several sentences that contain 25 ‘idea units’ (consisting of a target word, phrase, or idea) were read aloud to the participant who was then asked to retell each story immediately using the following instructions: ‘tell the story back to me, using as close to the same words as you can remember; you should tell me all you can, even if you are not sure’. The participant was then asked to recall both stories again after a 20–30 min delay. During their recanting the story, the examiner underlined correctly expressed idea units, and notated alternative wordings for later scoring.^18^ The delayed story recall total was included in this analysis as one of the two cognitive outcomes (possible range = 0 to 50); the other outcome was the total number of proper names produced in delayed recall. In order to create the proper names total scores, we used a previously described method where a database of idea units coded as separate variables were run through a part of speech tagger to assign each idea unit to lexical categories (e.g. proper names, verbs, numerical expressions, nouns); we then extrapolated a composite score of correctly recalled proper names in the delayed recall portion of the logical memory (0–9 correctly recalled proper names).^18^

### Cognitive status determination

To determine cognitive status after each visit, a ‘flagging algorithm’ based on a robust normative approach was applied to participants’ neuropsychological test scores.^20^ This algorithm ‘flags’ participants who are declining 1.5 SDs below the internal, robust normative means. WRAP researchers developed these norms in which normative distributions for cognitive test scores are made adjusting for age, sex, and literacy, where the normative group is non-declining over time.^21^ Records that were not flagged as potentially abnormal were then assigned a cognitive status of cognitively unimpaired—stable. Records that were flagged by the algorithm were then examined by a consensus review team, which then assigned the participant as either cognitively unimpaired-stable, cognitively unimpaired-declining, and MCI or dementia (based on NIA-AA criteria), and without reference to biomarkers.^20-22^

### Pittsburg compound B

The subset of the WRAP longitudinal cohort we selected for all analyses had undergone at least one 70 [^11^C]Pittsburgh compound-B (PiB) scan on a Siemens EXACT HR + scanner (and prior to 2015, a T1-weighted magnetic resonance imaging scan on a GE 3.0T MR750 using an 8-channel head coil). [^11^C]PiB radiosynthesis, acquisition and reconstruction parameters, image processing and quantification have been described previously.^23^ PiB positivity was ascertained by applying a threshold to the mean DVR (global PiB) across eight bilateral ROIs (global DVR > 1.19).^24^

### Statistical analysis

Statistical analyses were performed in R Version 4.2.2. Significance level was set at *P* < 0.05 across all analyses. To characterize how groups differed across sample characteristics, χ^2^ was used for categorical variables and Kruskal–Wallis for total years of education.

**Aim 1**: Sex as a moderator of age on Logical Memory measures over time

Linear mixed-effects regression was used to model the effect of sex on longitudinal decline on LMT delayed total scores and PN delayed scores. To facilitate interpretation of the models, all LMT and PN scores were first converted to *z* scores using the mean and standard deviation from the baseline visit of a subset of participants who were cognitively unimpaired at baseline (*n* = 449). Models were run using ‘lmer’ in the R package ‘lme4’.^25^ We included the random effect of subject-specific intercept in models to account for within-person correlations.^26^ Covariates for all models included age at visit, a practice effect term, a binarized education term, and standardized WRAT-3 reading scores (reflecting literacy).^27^ Wide range achievement test-3 was used to measure quality of education as it has been shown to reduce effects of social determinants of health discrepancies in education between ethnic and regional groups.^28^ The binarized education year term classified participants into two groups: those with 16 or more years of education (equivalent to Bachelor's Degree) and those with <16 years of education. Age was used as the time variable to account for varying time intervals between visits and the sex*age interactions were of primary interest in Aim 1.

**Aim 2:** Sex/PiB status group differences in change in longitudinal measures of logical memory

To examine the relationship between sex, PiB positivity, and age on logical memory delayed total scores and proper names delayed total scores, we again used linear mixed-effects models, replacing the sex*age interaction in the Aim 1 models with a 4− level sex/PiB status*age interaction where the four groups were female/PiB positive (*n* = 90), female/PiB negative (*n* = 222), male/PiB positive (*n* = 37), and male/PiB negative (*n* = 108). Significant interactions were followed by simple age slopes characterization and pairwise contrasts to identify which groups differed in slopes. Effect sizes were calculated for each pairwise comparison using Cohen's d.

### Sensitivity analyses

#### Pittsburgh compound B positivity threshold

Using a published equation, the validated local PiB DVR threshold of 1.19 corresponds to a Centiloid (Cl) of ∼21.6.^29^ While this corresponds to an increasingly commonly used cut-off of 20 CL, our data and others have shown that lower Cl thresholds (∼10–13 CL) include those who are in the most early stages of amyloid accumulation.^30-33^ Thus, in sensitivity analyses, we ran the above analyses at a lower mean PiB DVR (global PiB) threshold using global DVR > 1.13 (CL∼12.6).

## Results

**Aim 1:** Sex as a moderator of age on logical memory measures over time

Participant demographics and baseline clinical characteristics for Aim 1 are presented in Table 1 overall and by sex. A total of 457 participants with PiB PET imaging data, including 312 (68.27%) females, were included. Males were older and had more education; there were no other sex-specific differences in demographic or clinical characteristics.

**Table 1:** Participant demographics and baseline clinical characteristics: Aim 1

	Overall	Female	Male	*P*
*N* (%)	457 (100)	312 (68.27)	145 (31.72)	
Age at baseline logical memory (mean (SD))	58.15 (6.78)	57.78 (6.65)	58.95 (6.99)	0.086
Age at most recent visit (mean (SD))	67.07 (7.22)	66.56 (7.06)	68.17 (7.46)	**0.027**
Number of logical memory assessments (median [range])*	5 [1–7]	5 [1–7]	5 [1–7]	0.243
Race (*n* (%))				0.934
Non-Hispanic White	429 (93.9)	292 (93.6)	137 (94.5)	
African-American	21 (4.6)	15 (4.8)	6 (4.1)	
Hispanic	7 (1.5)	5 (1.6)	2 (1.4)	
Total years of education (median [Q1-Q3])	16 [14–18]	16 [14–17]	17 [16–18]	**<0.001**
WRAT-3 reading standard score (mean (SD))	106.44 (9.37)	105.98 (9.25)	107.43 (9.58)	0.122
Cognitive status at logical memory baseline (*n* (%))				0.104
Cognitively unimpaired—stable	380 (83.2)	265 (84.9)	115 (79.3)	
Cognitively unimpaired—declining	69 (15.1)	44 (14.1)	25 (17.2)	
Clinical MCI	8 (1.8)	3 (1.0)	5 (3.4)	
Parental history Alzheimer's disease dementia (*n* (%))	345 (75.5)	237 (76.0)	108 (74.5)	0.824
*APOE-ε4* carriers (*n* (%) 1 + alleles)	170 (37.2%)	119 (38.1%)	51 (35.2%)	0.680
PiB + (*n* (%))	127 (27.8)	90 (28.8)	37 (25.5)	0.531
Cognitive performance at logical memory baseline				
Logical memory total delayed recall (range 0–50) (mean (SD))	26.26 (7.10)	26.68 (6.77)	25.34 (7.69)	0.061
Proper names (range 0–9) (mean (SD))	4.87 (2.17)	5.01 (2.18)	4.58 (2.11)	0.05
MMSE (mean [median])	29.43 [30]	29.45 [30]	29.40 [30]	0.618

*‘Number of logical memory assessments’ reflects the total number over the full duration of the study. Bolded values indicate statistically significant results (*P* < 0.05).

WRAT-3, wide range achievement test-3 reading subtest; MMSE, mini-mental status examination; Logical memory, subtest from the Wechsler memory scale-revised (WMS-R).

**Figure 2:**
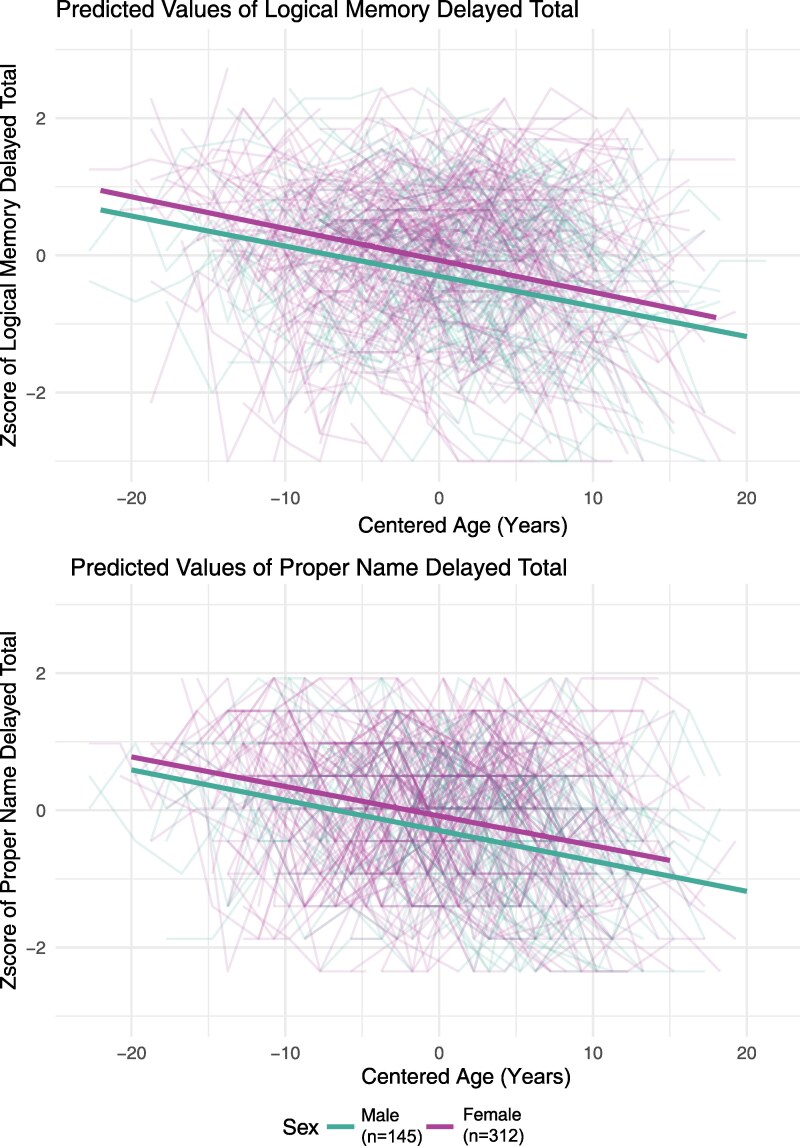
**Linear mixed-effect regression results: Aim 1.** The top panel shows predicted values of logical memory total score over time (*x*-axis) and the bottom panel depicts proper name recall over time. Age (years) is the time variable, and was centred on the mean age of 62.74 for ease of interpretation. The purple line represents females and the green line represents males. The lighter coloured lines in the figure represent each participant's data points over time (spaghetti plot). Lines were determined by linear mixed-effects regression models: outcome ∼ sex (female) + education (< = bachelor's degrees) + practice effects (nvis-1) + WRAT-III standard reading score + age + sex*age.

Results of the linear mixed-effect regression analysis of logical memory delayed total score and proper names delayed total score are presented side by side in Table 2 and graphically in Fig. 2 (with predicted male and female simple age slopes superimposed on spaghetti plots of individual performances). For both outcomes, the significant age terms (β = −0.04, *P* < 0.001 and β = −0.04, *P* < 0.001, respectively) and non-significant sex*age interactions (*P* = 0.713 and *P* = 0.871, respectively) indicated that both sexes experienced a similar average annual decline using age as the time variable. Female participants scored higher than males at baseline (coefficient = 0.23) on logical memory delayed total and on proper names delayed total (coefficient = 0.21).

**Table 2:** Linear mixed-effect regression results: Aim 1

	Z score of logical memory delayed total			Z score of proper names delayed total		
Predictors	Estimates	CI	*P*	Estimates	CI	*P*
(Intercept)	−4.46	−5.44 to −3.49	**<** **0.001**	−3.67	−4.55 to −2.79	**<0.001**
Sex (female)	0.23	0.06–0.40	**0.007**	0.21	0.06–0.37	**0.007**
Education (> = BA)	0.18	−0.00–0.37	0.053	0.10	−0.06–0.27	0.221
Practice effects	0.11	0.07–0.14	**<0.001**	0.05	0.02–0.09	**0.002**
WRAT-3 reading standard score	0.04	0.03–0.05	**<0.001**	0.03	0.02–0.04	**<0.001**
Age (centred)	−0.04	−0.06 to −0.03	**<0.001**	−0.04	−0.06 to −0.03	**<0.001**
Sex (female) × age	−0.00238	−0.02–0.01	0.713	0.00113	−0.01–0.01	0.871
Random effects						
σ^2^	0.36			0.46		
τ_00_	0.62_WRAPNo_			0.46_WRAPNo_		
ICC	0.63			0.50		
*N*	457_WRAPNo_			457_WRAPNo_		
Marginal *R*^2^/conditional *R*^2^	0.175/0.696			0.149/0.576		

*Practice effects* are calculated by subtracting the baseline visit from the total number of visits.^29^ Age was centred on the mean age of 62.74. Bolded values indicate statistically significant results (*P* < 0.05).

WRAT-3 reading, wide-range achievement test.

**Aim 2:** Sex/PiB status group differences in change in longitudinal measures of logical memory Participant demographics and clinical characteristics for Aim 2 are presented in Table 3 by the four sex/PiB status groupings: female PiB positive (*n* = 90), female PiB negative (*n* = 222), male PiB positive (*n* = 37), and male PiB negative (*n* = 108). Female PiB positive and male PiB positive groups differed from the female PiB negative group in both age at baseline and age at most recent visit. The female PiB negative group differed from the male PiB negative group in total years of education. The PiB positive groups differed from the PiB negative groups in *APOE* genotype. Sex/PiB status groups did not significantly differ in either number of visits or time between visits.

**Table 3:** Participant demographics and clinical characteristics: Aim 2

	Overall	Female PiB−	Female PiB+	Male PiB−	Male PiB+	*P*
*N* (%)	457	222 (48.57)	90 (19.39)	108 (23.63)	37 (8.1)	
Age at baseline logical memory (mean (SD))	58.15 (6.78)	57.05 (7.00)^1,2^	59.60 (5.33)^1^	58.39 (7.49)	60.59 (5.01)^2^	**0.002**
Age at most recent visit (mean (SD))	67.07 (7.22)	65.67 (7.25)^3,4^	68.77 (6.07)^3^	67.39 (7.64)	70.43 (6.49)^4^	**<0.001**
Number of logical memory assessments (median [range])	5 [1–7]	5 [1–7]	5 [1–7]	5 [1–7]	6 [1–7]	0.456
Time between baseline and most recent LM follow-up visit (mean (SD))	8.95 (3.84)	8.72 (3.69)	9.20 (3.78)	8.91 (4.19)	9.84 (3.75)	0.366
Race (*n* (%))						0.797
Non-Hispanic White	429 (93.9)	205 (92.3)	87 (96.7)	102 (94.4)	35 (94.6)	
African-American	21 (4.6)	13 (5.9)	2 (2.2)	5 (4.6)	1 (2.7)	
Hispanic	7 (1.5)	4 (1.8)	1 (1.1)	1 (0.9)	1 (2.7)	
Total years of education (median [Q1-Q3])	16 [14–18]	16 [14–17]^5^	17 [14–18]^6^	17 [16–18]^5,6^	17 [14–18]	**<0.001**
WRAT-3 reading standard score (mean (SD))	106.44 (9.37)	105.44 (9.33)	107.30 (8.95)	108.02 (9.13)	105.73 (10.74)	0.087
Cognitive status at logical memory baseline (*n* (%))						0.397
Cognitively unimpaired—stable	380 (83.2)	189 (85.1)	76 (84.4)	87 (80.6)	28 (75.7)	
Cognitively unimpaired—declining	69 (15.1)	30 (13.5)	14 (15.6)	17 (15.7)	8 (21.6)	
Clinical MCI	8 (1.8)	3 (1.4)	0 (0.0)	4 (3.7)	1 (2.7)	
Parental history Alzheimer's disease dementia (*n* (%))	345 (75.5)	159 (71.6)	78 (86.7)	76 (70.4)	32 (86.5)	0.054
*APOE-ε4* carriers (n (%) 1 + alleles)	170 (37.2%)	62 (27.9%)	57 (63.3%)	26 (24.1%)	25 (67.6%)	**<0.001**
Cognitive performance at logical memory baseline						
Logical memory delayed recall total score (range 0–50) (mean (SD))	26.26 (7.10)	26.31 (6.76)	27.60 (6.75)	25.45 (7.48)	25.03 (8.38)	0.125
Proper names delayed recall total score(range 0–9) (mean (SD))	4.87 (2.17)	5.00 (2.14)	5.03 (2.30)	4.70 (2.15)	4.22 (1.95)	0.153
MMSE (mean [median])	29.43 [30]	29.45 [30]	29.42 [30]	29.46 [30]	29.22 [30]	0.499

WRAT-3, wide range achievement test-3 reading subtest; MMSE, mini-mental status examination; Logical Memory, subtest from the Wechsler memory scale-revised (WMS-R).

*Post hoc* Tukey's test pairwise differences, confidence interval set at 0.95: baseline LM age 1: female PiB^−^<female PiB^+^  *P* = 0.01, 2: female PiB^−^<male PiB^+^  *P* = 0.02; age at most recent visit 3: female PiB^−^<female PiB^+^  *P* < 0.01, 4: female PiB^−^<male PiB^+^  *P* < 0.001; years of education 5: male piB^−^<female PiB^−^  *P* < 0.001, 6: male PiB^−^<female PiB^+^  *P* = 0.03. Bolded values indicate statistically significant results (*P* < 0.05).

**Aim 2a:** Sex/PiB status group differences in change in logical memory delayed recall

**Figure 3:**
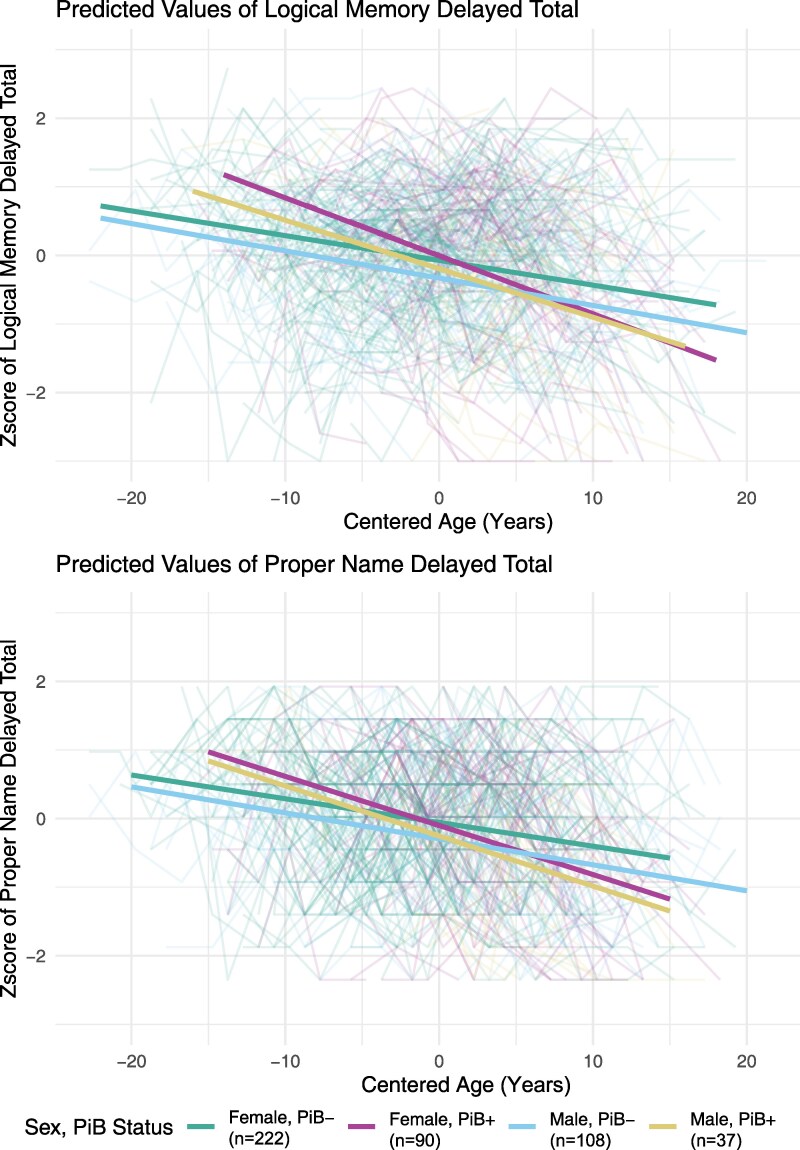
**Linear mixed-effect regression results: Aim 2.** The top panel shows predicted values of logical memory total score (*y*-axis) over time (*x*-axis) and the bottom panel depicts proper name recall (*y*-axis) over time (*x*-axis). Age (years) is the time variable, and was centred on the mean age of 62.74 for ease of interpretation. The green line represents females who are amyloid negative as measured by Pittsburgh compound-B, the purple line represents females who are amyloid positive; the light blue line represents males who are amyloid negative, and the yellow line represents males who are amyloid positive. The lighter coloured lines in the figure represent each participant’s data points over time (spaghetti plot). Lines were determined by linear mixed-effects regression models. Outcome ∼ sex (female) + education (< = bachelor’s degrees) + practice effects (nvis-1) + WRAT-III standard reading score + age + 4-level sex and amyloid status *age.

Results of the linear mixed-effect regression analysis of logical memory delayed total score are presented in Table 4 and graphically in Fig. 3. There was a significant sex/PiB status * age interaction in the PiB positive groups, indicating that PiB positivity was associated with faster decline in total score in both sexes. Specifically, simple age-slopes for change in total score were −0.0845 (−0.102, −0.0668) for female PiB + and −0.0361 (−0.0492, −0.0229) for female PiB−, and −0.0707 (−0.0941, −0.0473) for male PiB + and −0.0398 (−0.0551, −0.0245) for male PiB−. Pairwise contrasts indicated significant differences when comparing simple age-slopes of female PiB− and female PiB + (*P* < 0.0001, Cohen's *d* = 0.082), female PiB− and male PiB + (*P* = 0.0024, Cohen's *d* = 0.058), male PiB− and male PiB + (*P* = 0.0106, Cohen's *d* = 0.052), and female PiB + and male PiB− (*P* < 0.0001, Cohen's *d* = −0.075). Pairwise contrasts did not indicate significant differences in the simple age-slopes of female PiB− and male PiB− (*P* = 0.6210, Cohen's *d* = 0.006) or female PiB + and male PiB + (*P* = 0.2758, Cohen's *d* = −0.023).

**Table 4:** Linear mixed-effect regression results: Aim 2

	*Z* score of logical memory delayed total			*Z* score of proper names delayed total		
Predictors	Estimates	CI	*P*	Estimates	CI	*P*
(Intercept)	−4.24	−5.21 to −3.28	**<0.001**	−3.43	−4.30 to −2.55	**<0.001**
WRAT-3 reading standard Score	0.04	0.03–0.05	**<0.001**	0.03	0.02–0.04	**<0.001**
Education (> = BA)	0.20	0.01–0.38	**0.034**	0.12	−0.05–0.28	0.172
Practice effects	0.12	0.08–0.15	**<0.001**	0.06	0.03–0.09	**<0.001**
Age (Centred)	−0.04	−0.05 to −0.02	**<0.001**	−0.03	−0.05 to −0.02	**<0.001**
Female PiB+	0.07	−0.14–0.28	0.532	−0.04	−0.23–0.15	0.649
Male PiB−	−0.26	−0.46 to −0.06	**0.010**	−0.24	−0.42 to −0.06	**0.009**
Male PiB+	−0.12	−0.42–0.18	0.426	−0.20	−0.47–0.07	0.155
Age × Female PiB+	−0.05	−0.06 to −0.03	**<0.001**	−0.04	−0.05 to −0.02	**<0.001**
Age × Male PiB−	−0.0037	−0.02–0.01	0.619	−0.0033	−0.02–0.01	0.681
Age × Male PiB+	−0.03	−0.06 to −0.01	**0.002**	−0.04	−0.06 to −0.01	**0.003**
Random effects						
σ^2^	0.35			0.45		
τ_00_	0.61_WRAPNo_			0.46_WRAPNo_		
ICC	0.63			0.50		
*N*	457_WRAPNo_			457_WRAPNo_		
Marginal *R*^2^/conditional *R*^2^	0.186/0.701			0.159/0.581		

*Practice effects* are calculated by subtracting the baseline visit from the total number of visits; *WRAT-3 Reading*: wide-range achievement test.^29^ Age was centred on the mean age of 62.74. Regarding sex/PiB status: the 4- level sex/PiB status*age interaction where the four groups were female/PiB positive (*n* = 90), female/PiB negative (*n* = 222), male/PiB positive (*n* = 37), and male/PiB negative (*n* = 108) is a created variable to investigate Aim 2 and is not an interaction term. The values in this table are being compared to the female/PiB negative group. Bolded values indicate statistically significant results (*P* < 0.05).

**Aim 2b:** Sex/PiB status group differences in change in proper name recall

Results of the linear mixed-effect regression analysis of proper names delayed total score are presented in Table 4 and graphically in Fig. 3. For proper names delayed total score, there was a significant sex/PiB status*age interaction in PiB positive groups, indicating that PiB positivity was associated with faster decline in total score in both sexes. Simple-age slopes for change in total score were −0.0716 (−0.0898, −0.0533) for female PiB + and −0.0346 (−0.0473, −0.0219) for female PiB−, and −0.0730 (−0.0983, −0.0476) for male PiB + and −0.0379 (−0.0531, −0.0227) for male PiB−. Pairwise contrasts indicated significant differences when comparing simple age-slopes of female PiB− and female PiB + (*P* = 0.0001, Cohen's *d* = 0.055), female PiB− and male PiB + (*P* = 0.0027, Cohen's *d* = 0.057), male PiB− and male PiB + (*P* = 0.0097, Cohen's *d* = 0.052), and female PiB + and male PiB− (*P* = 0.0010, Cohen's *d* = −0.05). Pairwise contrasts did not indicate significant differences in the simple age-slopes of female PiB− and male PiB− (*P* = 0.6829, Cohen's *d* = 0.005) or female PiB + and male PiB + (*P* = 0.9921, Cohen's *d* = 0.002).

### Sensitivity analyses

#### Pittsburgh compound B positivity threshold

Linear mixed-effects regression was performed to examine differences in rates of decline between the four groups on logical memory delayed total score and proper names delayed total score using a lower PiB threshold (global DVR < 1.13). Participant demographics and sample characteristics are available in Supplementary Table 1 by the four new sex/PiB status groupings created by the lower threshold: female PiB positive (*n* = 113), female PiB negative (*n* = 199), male PiB positive (*n* = 45), and male PiB negative (*n* = 100). Results are available in Supplementary Table 2 and Supplementary Fig. 1. When lowering the PiB threshold, the overall patterns from the main analyses remained with minor differences that did not affect significance or conclusions.

## Discussion

In this investigation, we examined longitudinal trajectories of story recall tasks and whether sex-specific differences (Aim 1) or sex and combined amyloid status differences were present (Aim 2). Story recall was represented by the commonly used WMS-R logical memory delayed total score and a novel composite index of proper name recall. Our Aim 1 findings showed a main effect, such that female participants outperformed males on both the logical memory delayed total score and the proper names delayed score. Both sexes experienced a similar rate of decline over time, as we hypothesized. Our Aim 2 analyses showed that amyloid status was associated with faster rate of decline in proper name recall and total score recall for both sexes, such that amyloid positive males and females declined more rapidly compared to amyloid negative males and females. Although the difference in amyloid-related rate of decline between the sexes was not statistically significant, the effect size for total score in amyloid positive females was slightly larger than that of amyloid positive males, however both effect sizes were small (0.08 for females, 0.05 for males). Finally, contrary to our hypothesis, we found that male and female participants who were amyloid positive declined at ostensibly the same rate on proper names delayed recall score.

### Female advantage in logical memory proper name delayed recall

Our finding of a female advantage in story recall total score trajectories is in line with a 2023 meta-analysis of 496 effect sizes and 355 173 participants, showing a small but robust effect of a female advantage in episodic memory (delayed recall) across the lifespan.^34^ However, we also found a female advantage in proper name delayed recall. Previous studies on proper name retrieval, most often through face-name learning and memory tasks, have had conflicting results regarding whether or not sex differences exist.^35-37^ This novel finding in a relatively large sample (*n* = 457) with nearly 10 years of longitudinal follow-up suggests that, similar to other verbal learning and memory tasks, females may encode and retrieve proper names better than men. Some normative studies of proper name stimuli have been conducted, but none of these prior studies examined sex as a demographic variable.^38,39^ Prior theories about the mechanisms of memory for proper names suggests that successful encoding and retrieval is likely related to spatial, temporal, social, personal, and affective characteristics and experience.^40^ Proper name recall has been shown to decline in typical aging, with steeper declines in the presence of cognitive impairment.^36,41,42^ Further, previous research has demonstrated an age-related increase in tip-of-the-tongue errors, and a decrease in the proportion of correct responses for proper names than for object names.^43,44^ Most proposed explanations for the proper name difficulty centre on the idea that proper names can be considered ‘referring expressions’, which designate individual features rather than conceptual categories, and therefore do not have the same network of semantically related attributes to aid in retrieval that regular nouns do; how these features may influence encoding and retrieval by sex has not been investigated.^18,45^ Future longitudinal studies designed to examine sex differences using a wider range of proper names can help elucidate underlying neuropsychological and neurobiological mechanisms of these differences.

### Amyloid-beta-related decline in story recall delayed total score or proper name delayed recall does not differ by sex

We found that amyloid positive participants declined faster in total score delayed recall, but these trajectories did not differ by sex. Similar to these findings, association of accumulation of Aβ plaque and worsening performance on tests of story recall have been demonstrated in slightly older pre-clinical cohorts with different tracers.^46,47^ However, other studies have shown differing performance on tests of verbal memory between sexes in the presence of high Aβ deposition, which we did not see in our analyses as we had hypothesized.^14,48^ The sex by amyloid differences found in other studies have not been investigated on the logical memory subtest of the WMS-R, and instead used a common measure of verbal memory, the Rey Auditory Verbal Learning Test, and may be one reason for our discrepant findings. It is possible that the nature of the task—hearing a story and telling it back in conversation—is a more ecologically valid measure than list-learning, and that females may retain their advantage in the presence of amyloid accumulation, thus demonstrating cognitive resilience. Moreover, larger genome-wide studies have identified a genetic architecture of resilience, with several sex-specific molecular pathways underlying resilience.^49^ Other possible reasons for sex-specific resilience are not thoroughly understood, with some theories suggesting hormonal or structural brain differences between sexes as well as differences in cultural and social experience.^50-52^ Further study into the functional and structural differences in participants with high amyloid burden is needed to understand the underlying mechanisms of cognitive resilience across a range of neuropsychological measures. Furthermore, longer longitudinal follow-up with a wider range of impairment and amyloid burden may reveal additional sex-specific patterns.

We did not observe sex differences in amyloid-related decline in proper name recall; that is, pairwise contrasts revealed that amyloid positive males and amyloid positive females declined at a similar rate in proper name recall. The literature regarding sex differences in proper name recall shows that females perform better than males on the face-name associative memory exam in early midlife, but this advantage is attenuated in advanced age, suggesting a possible connection between memory function and hormone changes.^18^ Because the range of possible proper name items is relatively small (0–9), and because the WRAP sample is relatively young and unimpaired, it is possible that we did not have the range to detect sex differences at this time point; no other longitudinal study of this nature has examined sex differences in proper name trajectories in individuals with pre-clinical Alzheimer's disease. Future research in WRAP should include additional analyses of proper names stratified by sex and Alzheimer's disease biomarkers with additional time points of follow-up.

While previous research, as well as the present study, confirms that individuals with amyloid positivity tend to produce fewer proper names, our study demonstrates it is possible that sex does not further influence this relationship.

To our knowledge, this is the first study to investigate whether sex differences exist in proper name recall trajectories, and one of the first to examine whether sex differences exist in amyloid-related decline in proper names. Our novel finding that there is a female advantage in proper name recall further reinforces the sex-specific advantage females possess in verbal learning and memory. Previous research has shown that proper name recall is sensitive to Aβ deposition in unimpaired adults at risk for Alzheimer's disease, and the present study shows that the rate of amyloid-related decline does not differ by sex.^18,21,53^

A need for sex based normative standards on diagnostic test batteries has been previously proposed, and has been posited as an essential tool in the early detection of pre-clinical Alzheimer's disease in the female population.^17^ Prior research by our group included sex in the development of cross-sectional and longitudinal norms for several tests in the WRAP battery and for tests and composites harmonized between WRAP and the local Alzheimer's Disease Research Study.^52,54^ The latter includes norms for Logical Memory Story A (immediate and delayed recall). Future research may add similar norms for the proper names variables.^55^ Proper name recall has been previously demonstrated to be a better predictor of Aβ status than other story recall scores.^18,51^ A diagnostic tool that can circumvent the purported sex-specific cognitive reserve would be beneficial to early detection in female populations by reducing the number of false-negatives that may arise in tests of verbal learning and memory.

### Study limitations

Our study's strengths include the longitudinal design, the evaluation of sex-specific differences in story recall and their relationship to amyloid positivity and the examination of a novel composite score from logical memory, proper names’. Additionally, our study makes use of a well-established cohort that may be larger than other studies considering the impacts of sex and amyloid burden.

Our study is not without limitations. First, our study design had the inclusion criteria of all participants having at least two time points of Logical Memory data in order to ensure robust longitudinal analyses; however, in so doing, we may have excluded individuals who were lost to follow-up after their baseline visit, introducing a selection bias. Future studies will therefore specifically examine missing data and the potential impact these may have on assessment of cognitive decline in relation to Alzheimer's disease biomarkers. An additional potential limitation of our study is the use of the WMS-R, which is an outdated version of this test. The current version, the WMS-IV, also has an LMT component to it, but the vocabulary in the stories differ. As the WRAP study is a longitudinal study, we continue to use the WMS-R for the sake of consistency. Future studies should consider different test forms and how these may or may not replicate our findings, as well as how these test versions compare to each other. Another potential limitation in our work is the possible issue of false-positive Aβ findings in amyloid PET imaging, as has been noted to occur in a case study of an individual with behavioural variant frontotemporal dementia, who was classified as ‘amyloid positive’.^56^ Although we tested different thresholds of Aβ positivity in this study, it is still possible that some individuals near the threshold are mischaracterized. Future directions may include evaluating Alzheimer's disease biomarkers on a continuous scale to account for these potential variations. Finally, the study sample is predominantly non-Hispanic white and highly educated, and therefore not representative of the general population. Taken together, results should be interpreted with caution and require replication.

We showed that there was a main effect such that females generally performed higher on both Logical Memory delayed total and proper names delayed total, and that amyloid burden served as a significant predictor of decline across both sexes. As the WRAP is a cohort comprised of highly educated, predominantly non-Hispanic white, Midwestern individuals, it is necessary to replicate this study on other groups to validate these findings.

## Supplementary Material

fcaf169_Supplementary_Data

## Data Availability

The datasets presented in this article are not readily available because data are available through a data request process. Requests to access the datasets should be directed to https://wrap.wisc.edu/data-requests/. Access will be granted to named individuals in accordance with ethical procedures governing the reuse of sensitive data. Additionally, requestors must meet the following condition to obtain access: completion of a formal data sharing agreement. All analyses were performed in R version 4.4.2, and code for these analyses is available in Supplementary material.
