# Supplementary material for: Sex differences in story recall decline in preclinical Alzheimer's disease
Source: Brain Commun. 2025 May 5;7(3):fcaf169. doi: 10.1093/braincomms/fcaf169 (PMC12086310; doi:10.1093/braincomms/fcaf169)
Supplement: fcaf169_Supplementary_Data [file fcaf169_supplementary_data.pdf]

Supplementary Table 1

	Overall	Female PiB-	Female PiB+	Male PiB-	Male PiB+	p
N (%)	457	199	113	100	45	
Age at Baseline logical memory (mean (SD))	58.15 (6.78)	56.63 (6.94)	59.81 (5.60)	58.39 (7.59)	60.20 (5.30)	<0.001
Age at most recent visit (mean (SD))	67.07 (7.22)	65.23 (7.18)	68.90 (6.22)	67.39 (7.92)	69.89 (6.07)	<0.001
Number of logical memory assessments (median [range])	5 [1-7]	5 [1-7]	5 [1-7]	5 [1-7]	5 [1-7]	0.577
Race (n (%))						0.743
Non-Hispanic White	429 (93.9)	183 (92.0)	109 (96.5)	94 (94.0)	43 (95.6)	
African-American	21 (4.6)	12 (6.0)	3 (2.7)	5 (5.0)	1 (2.2)	
Hispanic	7 (1.5)	4 (2.0)	1 (0.9)	1 (1.0)	1 (2.2)	
Total Years of Education (median [Q1-Q3])	16 [14-18]	16 [14-17]	17 [14-18]	17 [16-18]	17 [14-18]	<0.001
WRAT-3 Reading Standard Score (mean (SD))	106.44 (9.37)	105.20 (9.53)	107.35 (8.60)	107.99 (9.23)	106.20 (10.31)	0.062
Cognitive Status at logical memory Baseline (n (%))						0.394
Cognitively Unimpaired - Stable	380 (83.2)	170 (85.4)	95 (84.1)	80 (80.0)	35 (77.8)	
Cognitively Unimpaired - Declining	69 (15.1)	26 (13.1)	18 (15.9)	17 (17.0)	8 (17.8)	
Clinical MCI	8 (1.8)	3 (1.5)	0 (0.0)	3 (3.0)	2 (4.4)	
Parental history AD dementia (n (%))	345 (75.5)	144 (72.4)	93 (82.3)	69 (69.0)	39 (86.7)	0.125
APOE-e4 carriers (mean (SD))	0.38 (0.49)	0.28 (0.45)	0.58 (0.50)	0.25 (0.44)	0.61 (0.49)	<0.001
Cognitive Performance at logical memory Baseline						
Logical memory delayed recall total score (range 0–50) (mean (SD))	26.26 (7.10)	26.32 (6.80)	27.32 (6.70)	25.79 (7.17)	24.36 (8.74)	0.102
Proper Names delayed recall total score(range 0–9) (mean (SD))	4.87 (2.17)	5.04 (2.15)	4.96 (2.25)	4.75 (2.16)	4.20 (1.95)	0.114
MMSE (mean [median])	29.43 [30]	29.45 [30]	29.42 [30]	29.46 [30]	29.22 [30]	0.626

Abbreviations: WRAT-3= Wide Range Achievement Test-3 Reading Subtest; MMSE= Mini-Mental Status Examination; R-AVLT= Rey Auditory Verbal Learning Test; Logical Memory= subtest from the Wechsler Memory Scale-Revised (WMS-R).

Supplementary Table 2

	Zscore of logical memory delayed total			Zscore of proper names delayed total		
Predictors	*Estimates*	*CI*	*p*	*Estimates*	*CI*	*p*
(Intercept)	-4.19	-5.16 – -3.22	**<0.001**	-3.39	-4.26 – -2.51	**<0.001**
WRAT-3 Reading Standard Score	0.04	0.03 – 0.05	**<0.001**	0.03	0.02 – 0.04	**<0.001**
Education (>=BA)	0.19	0.01 – 0.38	**0.038**	0.11	-0.05 – 0.28	0.179
Practice Effects	0.11	0.08 – 0.15	**<0.001**	0.06	0.02 – 0.09	**0.001**
Age (Centered)	-0.03	-0.05 – -0.02	**<0.001**	-0.03	-0.05 – -0.02	**<0.001**
Female PiB+	0.02	-0.18 – 0.22	0.841	-0.07	-0.25 – 0.11	0.431
Male PiB-	-0.25	-0.46 – -0.04	**0.018**	-0.24	-0.43 – -0.05	**0.011**
Male PiB+	-0.24	-0.52 – 0.04	0.092	-0.27	-0.52 – -0.02	**0.036**
Age × Female PiB+	-0.04	-0.06 – -0.03	**<0.001**	-0.03	-0.05 – -0.01	**<0.001**
Age × Male PiB-	-0.01	-0.02 – 0.01	0.473	-0.00	-0.02 – 0.01	0.595
Age × Male PiB+	-0.03	-0.05 – -0.01	**0.007**	-0.03	-0.06 – -0.01	**0.006**
**Random Effects**						
σ^2	0.35			0.45		
τ_{00}	0.61 $_{\text{WRAPNo}}$			0.46 $_{\text{WRAPNo}}$		
ICC	0.63			0.50		
N	457 $_{\text{WRAPNo}}$			457 $_{\text{WRAPNo}}$		
Observations	2013			1982		
Marginal R^2 / Conditional R^2	0.185 / 0.701			0.158 / 0.580		

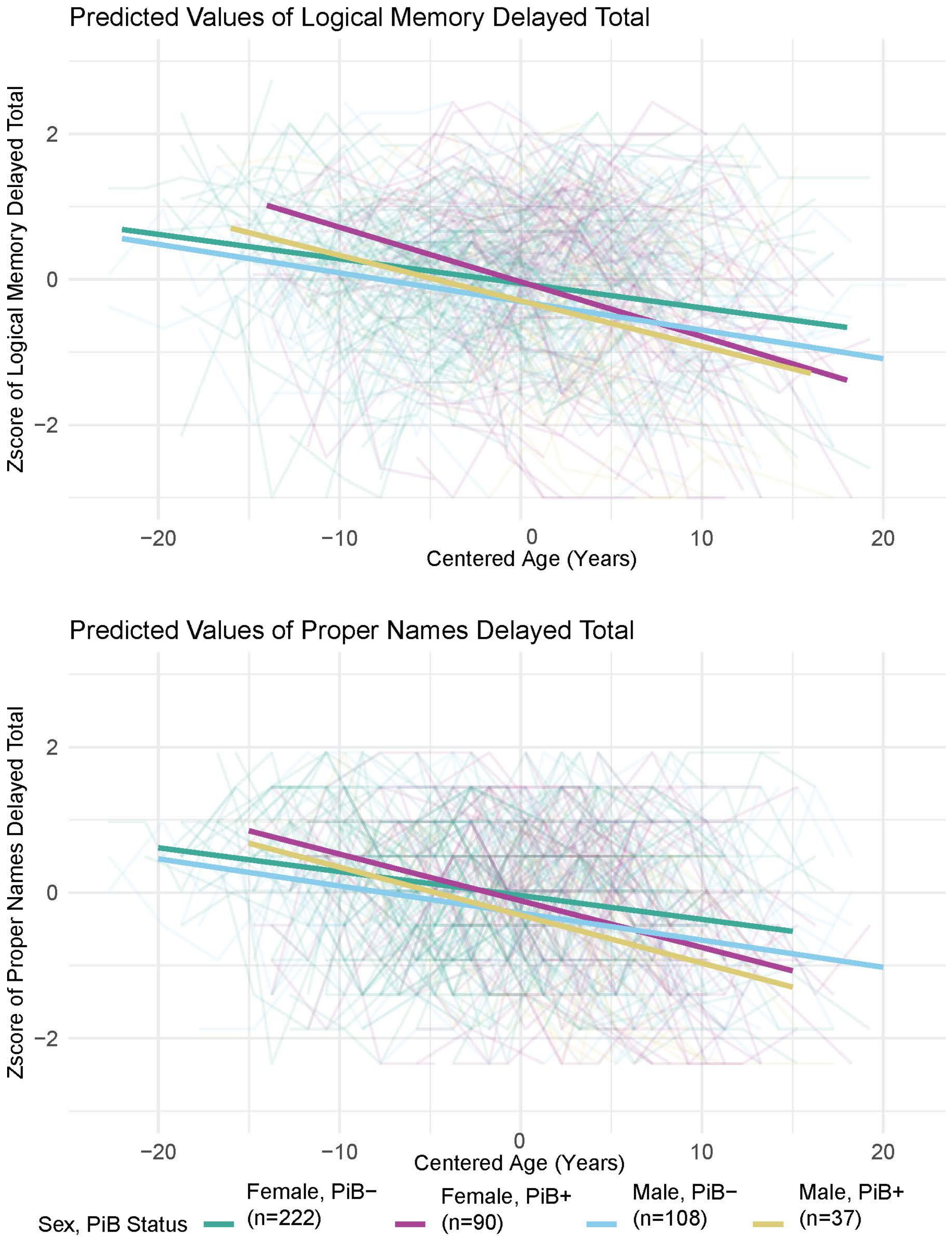

Supplemental Figure 1. Linear mixed effects regression models examining differences in rates of decline between 4 groups on Logical Memory delayed total score and proper names delayed total score using a lower PiB threshold (global DVR<1.13). The top panel shows predicted values of Logical Memory total score (y-axis) over time (x-axis) and the bottom panel depicts Proper Name recall (y-axis) over time (x-axis). Age (years) is the time variable and was centered on the mean age of 62.74 for ease of interpretation. The green line represents females who are amyloid negative as measured by Pittsburgh Compound-B, the purple line represents females who are amyloid positive; the light blue line represents males who are amyloid negative, and the yellow line represents males who are amyloid positive. The lighter colored lines in the figure represent each participant's data points over time (spaghetti plot). Lines were determined by linear mixed effects regression models. Outcome ~ sex (female) + education (<=Bachelor's degress) + Practice effects (nvis-1) + WRAT-III standard reading score + age + 4-level sex & amyloid status *age.

R-code for “Sex differences in Story Recall decline in Preclinical Alzheimer’s Disease”

Kimberly D. Mueller, Ph.D., University of Wisconsin - Madison

2024-12-05

Introduction

This is code for the analyses in Cowman et al., “Sex differences in Story Recall decline in Preclinical Alzheimer’s Disease”. All analyses were performed in R version 4.4.2 (2024-10-31) -- "Pile of Leaves" Copyright (C) 2024 The R Foundation for Statistical Computing

Load Libraries

##Create 4 level pib_gender variable (A+/- Female, A+/- Male)

```
set2$pib_gender <- ifelse(set2$gender == 1 & set2$pib_bin == 0, "Male_Neg",
                          ifelse(set2$gender == 1 & set2$pib_bin == 1,
"Male_Pos",
                                 ifelse(set2$gender == 2 & set2$pib_bin == 0,
"Female_Neg",
                                        "Female_Pos")))
```

##Aim 1: Sex as a moderator of age on logical memory measures

```
#Logical memory delayed total
q1 <- lmer(ZwmsrTot2 ~ gender + readstn + curage + (1|ID) + gender*curage,
data=set2, REML=FALSE)
mod1 <- ggpredict(model=q1, terms = c("curage", "gender"), group.terms =
c("gender"))
plot(mod1)
tab_model(q1)
ggpredict(model=q1, terms = c("curage", "gender"), group.terms = c("gender"))
plot(mod1) +
  geom_line(linewidth = 1)+
  coord_cartesian(xlim = c(60, 84))
#Proper names delayed total
q1b <-lmer(ZPropName_DelAB ~ gender + readstn + curage + (1|ID) +
gender*curage, data=set2, REML=FALSE)
mod1b <- ggpredict(model=q1b, terms = c("curage", "gender"), group.terms =
c("gender"))
plot(mod1b)
tab_model(q1b)
ggpredict(model=q1b, terms = c("curage", "gender"), group.terms =
```

```
c("gender"))
plot(mod1b) +
  geom_line(size = 1)+
  coord_cartesian(xlim = c(60, 85))
```

##Aim 2: Sex as a moderator of amyloid burden on logical memory measures over time

```
q2 <- lmer(ZwmsrTot2 ~ readstn + curage + (1|ID) + pib_gender*curage,
data=set2, REML=FALSE)
tab_model(q2)

library(emmeans)

# Calculate estimated marginal means and perform pairwise comparisons
emm <- emmeans(q2, ~ pib_gender | curage)
pairs(emm)


mod2 <-ggpredict(model=q2, terms = c("curage", "pib_gender"), group.terms =
c("pib_gender"))
plot(mod2) +
  geom_line(size = 1)+
  coord_cartesian(xlim = c(60, 85))
#Proper names delayed total
q2b <- lmer(ZPropName_DelAB ~ readstn + curage + (1|ID) + pib_gender*curage,
data=set2, REML=FALSE)
tab_model(q2b)

library(emmeans)

# Calculate estimated marginal means and perform pairwise comparisons
emm <- emmeans(q2b, ~ pib_gender | curage)
pairs(emm)



mod2b <-ggpredict(model=q2b, terms = c("curage", "pib_gender"), group.terms =
c("pib_gender"))
plot(mod2b) +
  geom_line(size = 1)+
  coord_cartesian(xlim = c(60, 85))
```
